# Violence and sexual and reproductive health service disruption among girls and young women during COVID-19 pandemic in Nepal: A cross-sectional study using interactive voice response survey

**DOI:** 10.1371/journal.pone.0260435

**Published:** 2021-12-08

**Authors:** Ashish Lamichhane, Shubheksha Rana, Krishna Shrestha, Rakshya Paudyal, Parash Malla, Shanti Upadhyaya, Durga Uprety, Julie Gurung, Elizabeth Satow

**Affiliations:** Plan International Nepal, Kathmandu, Nepal; Population Council, INDIA

## Abstract

**Introduction:**

There is a paucity of data on the impact of COVID-19 pandemic on girls and young women. The study examines the prevalence and correlates of violence and sexual and reproductive health (SRH) service disruption among girls and young women during COVID-19 restrictions and lockdown.

**Methods:**

An interactive voice response survey was conducted among girls and women aged 18–24 years between 10th March to 24th April 2021. The survey enrolled 1314 participants. Descriptive analysis was used to assess prevalence of violence and SRH service disruption. Two sampled test of proportion was used to asses difference in prevalence of violence before and after the pandemic. Logistic regression was used to examine relationship between the outcome variables and socio demographic predictors.

**Results:**

The study did not find significant difference in prevalence of violence before and after the pandemic. Education was found to be protective against experience of both physical and sexual violence after the pandemic. Dalit participants were four times more likely to report physical violence after the pandemic than Brahmin/Chhetri participants (OR:3.80; CI:1.41–10.24). Participants from 22-24-year age group were twice as likely to experience sexual violence compared to girls and participants from 18-21year age group (OR:2.25; CI:1.04–4.84). Participants from urban municipalities were 29% less likely to report SRH services disruption than participants from rural municipalities (OR-0.71, 95% CI: 0.55–0.91). Participants with disability were twice as likely to report disruption than participants without disability (OR-2.35, 95% CI: 1.45–3.82).

**Conclusions:**

To reduce violence against girls and women due to the pandemic, interventions should focus on Dalit women and on preventing education discontinuation among girls and women. SRH service during the pandemic needs to be improved for girls and women with focus on girls and women from rural municipalities and girls and women with disability.

## Introduction

The Coronavirus (COVID-19) pandemic has led to significant loss of lives worldwide. The pandemic has also led to substantial economic and social disruptions, with millions of people losing their livelihood (with risk of falling into extreme poverty) and experiencing disruption in essential services [1, 2].

While it’s evident that the impact of COVID-19 outbreak and lockdown has affected and will continue to affect all strata of the population, poor, marginalized, and socially excluded population are particularly vulnerable and are likely to be disproportionately affected. Young women and girls are known to be vulnerable to any crisis situation [3]. There have been reports of increased incidence of violence among girls and young women around the globe, likely exacerbated by economic strain [4, 5]. A crisis situation such as this is also likely to disrupt access to essential sexual and reproductive health services for girls and young women [3].

Gender based violence and limited access to sexual and reproductive health (SRH) services are two of the major public health issues faced by girls and young women in Nepal. Nepal Demographic and Health Survey (NDHS) [6] reported that 13% of girls and women aged 15–24 years’ experienced physical violence and 5% experience sexual violence in their lifetime.

Access to SRH services is challenging, especially in rural municipalities. The access of menstrual hygiene products is poor in rural municipalities due to difficult topography and affordability [7, 8]. The contraceptive prevalence rate of modern method among married women stands at 53% and unmet need for family planning is 24% [6]. This is attributed to poor reach of services in hard to reach communities and lack of adequate methods being distributed [9]. Similarly, there is a long-standing issue of poor HIV testing coverage in Nepal due to limited number of testing sites [9]. There is shortage of abortion medications in rural municipalities due to poor supply management [10].

With the current COVID-19 pandemic and lockdown, incidence of violence could increase and access to SRH services could be further affected. There are already reports and warnings from different agencies regarding rise of gender-based violence (GBV) and inaccessibility of SRH services among girls and women [11–13]. However, there is a paucity of data on actual magnitude and distribution of these issues in the population. This study thus seeks to assess the prevalence of physical and sexual violence before and after the COVID-19 pandemic, the extent of disruption in essential SRH services among adolescent girls and young women and identify associated predictors.

## Methods

Data collection was carried out through Interactive Voice Response (IVR) survey among adolescent girls and young women aged 18–24 years [14]. IVR survey is a data collection method that uses prerecorded questions to administer survey over telephone [15]. The method does not require live interviewers. Respondents enter their response by pressing buttons on the phone keypads. Their responses are then recorded and compiled automatically at the backend. The survey was conducted among mobile phone users. The survey used prerecorded questions available in top five spoken languages in Nepal [16]–Nepali, Maithali, Bhojpuri, Tharu and Tamang.

The sample size calculation was based on the analysis of interest. The study primarily sought to compare outcomes by place of dwelling—urban vs rural. A sample size of 600 was obtained per domain of interest, assuming 50% estimated prevalence, 95% confidence interval and 4% margin of error [17].

The participants were sampled using random digit dialing (RDD) method. The phone numbers were randomly generated and dialed. The generated number could belong to participants residing in any part of Nepal. The system ensured that same number was not dialed twice.

The questionnaire was pretested with the study demographic before data collection to inform content validity, comprehension and appropriateness. Data collection was carried out between 10th March and 24th April 2021. A total of 201,565 phone numbers were dialed, of which, 40,849 calls were connected. About 2,515 respondents met the study’s gender and age criteria (female aged between 18 to 24 years) and started the survey. The survey was stopped after obtaining 1314 completed responses (114 more samples than envisaged) with a completion rate of 52%.

### Ethical approval and consent

Informed consent was obtained through the IVR system. At the beginning of the survey a recording explaining study’s objective, voluntary nature of research, potential risks, benefits, use of data during analysis and rights of participants was played. After this, participants communicated their consent though their phone’s keypad. Research protocol and research questionnaire was approved by Nepal Health Research Council ***(Reg*. *863/2020P)*,** a national government body that regulates health research in Nepal.

### Measures

The operational definitions of the study variables are provided in Table 1.

**Table 1:** List of variables used in the analysis.

Outcome variables	
Experience of physical violence before COVID-19	Coded as 1 If participants reported at least one of following acts done to them before COVID-19 pandemic:
	(a) Slapped you or thrown something at you that could hurt you.
	(b)Pushed you or shoved you or pulled your hair.
	(c) Hit you with fist or with something else that could hurt you.
	(d)Kicked you, dragged you, beaten you up, choked you or burnt you on purpose.
	(e)Threatened to use or actually used a gun, knife or weapon against you.
Experience of physical violence after COVID-19	Coded as 1 if participant reported one of the acts above after the pandemic
Experience of sexual violence before COVID-19	Coded as 1 if participants reported at least one of the acts done to them before COVID-19 pandemic:
	(a) Physically forced you to have sexual intercourse with him even when you did not want to.
	(b)Forced you to have sexual intercourse when you were afraid to say ‘no’ to sex.
	(c)Forced you to do something sexual that you found degrading or humiliating.
Experience of sexual violence after COVID-19 and	Coded as 1 if participant reported one of the acts above after the pandemic
Experience of violence (physical or sexual violence) before COVID-19	Coded as 1 if participants reported a form of physical or sexual violence before the pandemic
Experience of violence (physical or sexual violence) after COVID-19	Coded as 1 if participants reported a form of physical or sexual violence after the pandemic
Experience of disruption in accessing SRH services due to COVID-19	Coded as 1 if participants reported barriers in accessing sexual and reproductive health services due to COVID-19 restrictions and lockdown. The services included access to menstrual hygiene products, modern or emergency contraceptives, safe abortion services, physician consultations, HIV and sexually transmitted infection testing and treatment or other services.
Predictor variables	
Age	Dichotomous variable; 18 to 21 years or 22 to 24 years
Place of residence	Dichotomous variable; urban or rural
Disability	Self-reported dichotomous variable; Yes or No
Ethnicity	(a) Dalit (untouchables, low caste), (b) Janajati (indigenous group), (c)Madhesi (group originally from Terai plain), (d)Muslim, (e) Newar (specific group of indigenous population), (f) Brahmin/Chhetri (upper caste) and (g) Other. Madhesi, Newar and Muslim category were grouped into ‘Other’ in regression models with violence outcome indicators due to low number of events across these categories (less than five)
Education	(a) No formal education, (b) some or completed primary, (c) some or completed secondary, (d) undergraduate and (e) postgraduate and above. Dichotomous variable (some formal education and no formal education) was used in regression models with violence outcome variable due to low variation in events across education categories.
Marital Status	Currently married participants were coded as ‘Married’ while participants who were divorced, unmarried or widow were coded as ‘Single’. Divorced and widowed participants were grouped into ‘Single’ due to low variation of outcomes in these categories (less than five)

The study assessed the experience of physical violence and sexual violence before and after the pandemic. The questions on the forms of violence were adapted from a 2005 WHO multi country study on women’s health and domestic violence against women [18]. Before participants responded to questions related to violence, they were informed what constituted physical or sexual violence in the study through a recording.

In order to assess disruption in SRH service, participants were asked if they experienced barriers in accessing SRH services. This included access to menstrual hygiene products, modern or emergency contraceptives, safe abortion services, physician consultations, HIV and sexually transmitted infection testing and treatment or other services.

Predictor variables included sociodemographic variables like age, place of residence, disability status, ethnicity, education and marital status. The ethnicity variable was adapted from Nepal’s Central Bureau of Statistic’s classification of main caste/ethnic groups [19].

### Analysis and diagnostic tests

Data was analyzed using Stata version 14. Two sample test of proportion was used to test the equality of proportion of participants reporting violence before and after the pandemic. Logistic regression was used to examine association between predictor and outcome variables. Huber White sandwich estimator was used to address heteroskedasticity in the regression models [20]. The models used odds ratio instead of coefficients since the direct interpretation of coefficients in a nonlinear regression is challenging [21]. Pseudo-R squared estimate were generated for each of the models. The estimate provides the extent of variation explained by the models in the outcome variable. Receiver Operating Characteristics, plot of true positive prediction rate against false prediction rate, was plotted for each model to assess the predictive accuracy of the models. The plot provides concordance index (c statistic). The c statistic ranges from 0 to 1, where value below 0.5 represents routine misprediction, 0.5 entails random prediction and 1 indicates perfect prediction of outcome [21].

The events per predictor variable for the regression models ranged from 5–63. The rule of thumb in a logistic regression is to have at least 10 events per variable [22]. However, there are evidence that the rule could be relaxed to 5–9 events per variable without incurring significant impact to the models [23].

## Results

The sociodemographic characteristics of the participants are presented in Table 2.

**Table 2:** Socio-demographic characteristics of the participants.

Predictor variables	Freq.	%
***Age group***		
between 18–21	804	61
between 22–24	510	39
***Ethnicity***		
Brahmin/Chhetri	611	47
Dalit	100	8
Janajati	379	29
Madhesi	74	6
Muslim	11	1
Newar	64	5
Other	75	6
***Place of residence***		
Rural municipality	583	44
Urban municipality	731	56
***Educational Status***		
No formal education	110	8
Some or completed primary	193	15
Some or completed secondary	362	28
Undergraduate	337	26
Postgraduate and above	312	24
***Disability (Y/N)***		
No	1231	94
Yes	83	6
***Marital Status***		
Divorced	15	1
Married	474	36
Unmarried	823	63
Widow	2	0
Total	1314	100

Around 6% of the participants reported experiencing a form of physical or sexual violence before the COVID-19 pandemic (Table 3). The proportion of participants reporting a form of violence decreased by around 1% after the COVID-19 pandemic. The proportion was higher in rural municipalities, both before and after the pandemic.

**Table 3:** Distribution of outcome variables by place of residence with 95% confidence interval.

Outcome variables	Rural (%)	Urban (%)	Total (%)
Experience of a form of violence *(Before COVID-19)*	6.2 (4.5–8.5)	5.3 (3.9–7.2)	5.7 (4.6–7.1)
Experience of a form of violence *(After COVID-19)*	6.3 (4.6–8.6)	3.01 (1.99–4.5)	4.5 (3.5–5.75)
Physical Violence *(Before COVID-19)*	4.5 (3.1–6.5)	3.6 (2.4–5.2)	3.95 (3.02–5.2)
Physical Violence *(After COVID-19)*	4.3 (2.8–6.3)	1.6 (0.8–2.8)	2.8 (2.04–3.9)
Sexual Violence *(Before COVID-19)*	2.4 (1.4–4.01)	2.2 (1.3–3.5)	2.3 (1.6–3.24)
Sexual Violence *(After COVID-19)*	3.3 (1.97–5)	1.4 (0.6–2.5)	2.2 (1.5–3.2)
SRH service disruption	33.8 (30.1–37.7)	24.5 (21.5–27.7)	28.6 (26.2–31.1)
***Total***	***583***	***731***	***1314***

Around 4% of participant reported experiencing physical violence before the pandemic compared to 3% after the pandemic. The experience of physical violence remained similar in rural municipalities while it decreased in urban municipalities by 2%.

Experience of sexual violence remained similar both before and after the pandemic at 2%. However, there was an increase of around 1% in the rural municipalities. There was a decrease of around 1% in the urban municipalities.

Around 29% of participants (382) reported facing difficulty accessing SRH services (Table 4). One third (34%) of the participants residing in rural municipalities reported disruption in accessing SRH service compared to one quarter (25%) residing in urban municipalities. More than half of them (52%) reported difficulty in accessing menstrual hygiene product. Around one in ten (10%) reported not having access to physician consultation. Similar proportion (9%) reported disrupted access to modern contraceptives. Similarly, around 8% reported disruption in accessing safe abortion service and 2% reported disrupted access to HIV/STI infection testing and treatment. Around 19% reported other disruptions.

**Table 4:** Reported disruption in SRH services.

Disrupted SRH services	Freq.	% Of cases
**Menstrual hygiene**	198	52
**Physician consultations**	39	10
**Modern/emergency contraception**	35	9
**Safe abortion service**	32	8
**HIV and sexually transmitted infection testing or treatment**	9	2
**Other**	73	19
**Total cases**	**382**	

### Multivariate analysis

Experience of a form of violence (physical or sexual), both before and after the pandemic was significantly associated with education (Table 5). Participants with no formal education were four times more likely to experience a form of violence than participants who had some formal education, both before (OR:3.94; CI:2.07–7.52) and after (OR:4.13; CI: 2.11–8.06) the pandemic. There was a significant association (p<0.01) between experiencing a form of violence and ethnicity after the pandemic. Participants from Dalit caste community were three times more likely to experience a form of violence than participants from Brahmin/Chhetri caste community (OR:3.28; CI: 1.50–7.16).

**Table 5:** Regression models looking at association between predictor socio demographic variables and experience of violence.

Predictor variables	Experience of violence (Before COVID-19)		Experience of violence (After COVID-19)		Experience of physical violence (Before COVID-19)		Experience of physical violence (After COVID-19)		Experience of sexual violence (Before COVID-19)		Experience of sexual violence (After COVID-19)	
	*Pseudo R2–0*.*0606 C statistic—0*.*6830*		*Pseudo R2–0*.*0887 C statistic—0*.*72*		*Pseudo R2–0*.*0284 C statistic—0*.*65*		*Pseudo R2- 0*.*0699 C statistic = 0*.*7165*		*Pseudo R2- 0*.*1185 C statistics—0*.*797*		*Pseudo R2–0*.*1023 C statistics—0*.*769*	
	Odds Ratio	P>z	Odds Ratio	P>z	Odds Ratio	P>z	Odds Ratio	P>z	Odds Ratio	P>z	Odds Ratio	P>z
***Residence***		** **		** **		** **		** **		** **		** **
Rural (reference)		** **		** **		** **		** **		** **		** **
Urban	1.07	0.80	0.60	0.08	0.92	0.77	0.48	0.05	1.19	0.65	0.52	0.11
***Age***		** **		** **		** **		** **		** **		** **
18 to 21 years (reference)		** **		** **		** **		** **		** **		** **
21 to 24 years	1.01	0.96	1.26	0.45	0.91	0.77	0.85	0.68	1.67	0.18	2.25	**0.04**
***Ethnicity***		** **		** **		** **		** **		** **		** **
Brahmin/Chhetri (reference)		** **		** **		** **		** **		** **		** **
Dalit	1.44	0.35	3.28	**0.00**	1.44	0.43	3.80	**0.01**	0.92	0.90	1.76	0.37
Janajati	1.06	0.85	1.45	0.28	1.16	0.65	1.87	0.17	0.78	0.58	1.16	0.74
Other*	0.46	0.07	1.22	0.62	0.53	0.19	1.97	0.16	0.41	0.17	0.76	0.62
***Education***		** **		** **		** **		** **		** **		** **
Some formal education (reference)		** **		** **		** **		** **		** **		** **
No formal education	3.94	**0.00**	4.13	**0.00**	1.88	0.18	2.61	**0.04**	11.85	**0.00**	6.89	**0.00**
***Person with Disability***		** **		** **		** **		** **		** **		** **
No		** **		** **		** **		** **		** **		** **
Yes	1.99	0.08	1.55	0.33	2.00	0.15	1.72	0.31	1.70	0.35	0.87	0.84
***Marital status***		** **		** **		** **		** **		** **		** **
Single (reference)		** **		** **		** **		** **		** **		** **
Married	1.41	0.21	1.56	0.15	1.53	0.21	1.40	0.40	0.59	0.22	1.04	0.92
_cons	0.04	**0.00**	0.03	**0.00**	0.03	**0.00**	0.02	**0.00**	0.01	**0.00**	0.01	**0.00**

* Madhesi, Newar and Muslim were grouped into others due to low number of events per category (less than 5).

Experience of physical violence before COVID-19 was not associated with any of the sociodemographic predictors. The experience of physical violence after COVID-19 was associated with education and ethnicity. Participants with no formal education were about three times more likely to experience physical violence after pandemic than participants with some formal education (OR:2.61; CI: 1.04–6.55). Dalit participants were four times more likely to report physical violence after pandemic than Brahmin/Chhetri participants (OR:3.80; CI: 1.41–10.24).

Experience of sexual violence before the pandemic was 12 times more likely among participants with no formal education (OR:11.85; CI: 5.27–26.63). After the pandemic, participants with no formal education were seven times more likely to experience sexual violence (OR:6.89; CI: 3.01–15.77). Similarly, participants from 22 to 24-year age group were twice as likely to experience sexual violence compared to participants from 18 to 21-year age group (OR:2.25; CI: 1.04–4.84).

Disruption to SRH services was significantly associated with place of residence and disability status(p<0.01). Participants from urban municipalities were more 29% less likely to report disruption than participants from rural municipalities (OR-0.71, 95% CI: 0.55–0.91) (Table 6). Similarly, participants with disability were twice as likely to report disruption than participants without disability (OR-2.35, 95% CI: 1.45–3.82). There was no association with ethnicity, education, age and marital status.

**Table 6:** Regression models looking at association between experience of SRH service disruption and sociodemographic variables.

Predictor variables	SRH service disruption			
	*Pseudo R2-0*.*0266*			
	*C statistic- 0*.*61*			
	Odds ratio	P>z	95% Confidence Interval	
***Residence***				
Rural (reference)				
Urban	0.71	**0.008**	0.55	0.91
**Age**				
18 to 21 years (reference)				
21 to 24 years	1.01	0.931	0.77	1.33
***Ethnicity***				
Brahmin/Chhetri (reference)				
Dalit	1.13	0.611	0.70	1.82
Janajati	1.09	0.558	0.81	1.46
Madhesi	1.48	0.135	0.88	2.49
Muslim	0.79	0.722	0.21	2.98
Newar	0.98	0.950	0.54	1.78
Other	1.10	0.739	0.64	1.88
***Education***				
No formal education (reference)				
Postgraduate and above	0.78	0.348	0.47	1.30
Some or completed primary	0.80	0.395	0.49	1.33
Some or completed secondary	0.82	0.411	0.51	1.32
Undergraduate	0.62	0.059	0.38	1.02
***Person With Disability***				
No				
Yes	2.35	**0.001**	1.45	3.82
***Marital status***				
Single (reference)				
Married	1.28	0.070	0.98	1.67
_cons	-0.69	0.005	0.31	0.81

## Discussion

The study seeks to document the prevalence and correlates of violence and SRH service disruption among adolescent girls and young women in Nepal in the backdrop of the global COVID-19 pandemic.

The study did not find a significant change in prevalence of physical or sexual violence among the participants. This is in contrast to studies and commentaries from several countries around the globe, not limited to low- and middle-income countries, that postulate increase in violence cases following the pandemic [24–33]. The assertions were based on anecdotal evidence, police reports, increased demand for emergency services, shelters, and calls or contacts with help services. In Nepal, a total of 885 complaints of domestic violence was reported in the helpline service operated by National Women Commission between April to June 2020. This was more than twice the number reported during the same period before COVID-19 [34]. In Rio de Janeiro, office of the state prosecutor reported 50% increase in domestic violence in the first weekend after statewide social distancing order [24]. Similarly a study of US police departments across four states reported an increase of 10 to 27% in domestic violence calls in March 2020 compared to March 2019 following lockdown orders in March [25]. World Health Organization Europe member states reported a 60% increase in emergency calls in April 2020 compared to the same period a year before [28]. The difference in results could be explained by the fact that this was a cross sectional study in the general population. The use of IVR survey was informed by the principle of do no harm, to reduce the risk of COVID-19 transmission associated with face-to-face interviews. However, the use of IVR survey for this study could have led to lower prevalence of violence in this study. Victims of violence are likely to have restricted means of communication with outside world [27, 35]. This might have created a barrier for some victims to take part in the study.

Education status was a protective factor against both form of violence after the pandemic. This association is supported by other pre-pandemic studies from Nepal [6, 36]. A study from Uganda attributed the decreased likelihood of violence in educated women to the delay in marriage and increased employment opportunities associated with higher education [37]. Both of the factors are likely to improve women’s agency and bargaining power in family as well as within marriage. Educated and economically independent women may be more vocal and less likely to conform to traditional gender roles [36]. Another possible explanation could be the fact that schools are considered to be a safe place. A study from Congo found that higher level of participation in formal education had protective effect on experience of physical and sexual violence [38]. In the study, school was deemed to be a ‘safe place’ by the girls.

Dalit participants were more likely to experience physical violence after the pandemic. This is in line with other pre-pandemic studies in Nepal [39, 40] Dalits in Nepal are a socially marginalized group and face discrimination in social, political, education, health and economic spheres. Dalit women are more likely to face violence due to their low social standing, poor socio economic status, lower literacy rate and higher vulnerability to early marriage [40, 41]. The COVID-19 pandemic has possibly amplified these factors, making Dalit women more vulnerable to physical violence.

Women from 21 to 24 years were more likely to experience sexual violence. This is consistent with the findings from the NDHS 2016 which found that sexual violence increased with age among women [6]. However there are studies from countries like Turkey and US, from before the pandemic, that contradicts these findings and find an opposite trend of association [42, 43]. These studies found that the prevalence of violence decreased with age. The association between age and sexual violence is complicated and possibly differs between cultures and setting. Age could have also served as a proxy for another variable not covered in the study. Further research is needed to understand the relationship between age and violence during pandemic.

The study found an association between disability and disrupted access to SRH services. The result is supported by a multi country study conducted by UNFPA that reported incidences of SRH service disruption during COVID-19 among women, girls and gender non-conforming persons with disability [44]. COVID-19 lockdowns and restrictions has affected SRH service access for all girls and women, but the disruption in service access are exacerbated for girls and women with disabilities [44]. This could be attributed to new protocols in health care and community setting that do not account for people with disability as well as preexisting barriers [44]. This includes lack of special equipment and facilities, stigma surrounding disability and inadequate training and negative attitude of health care providers [44].

Expectedly, the access to SRH services following the pandemic was poor in rural municipalities. Nepal has had challenges in ensuring SRH services in rural municipalities, attributed to difficult topography in rural municipalities, lack of funds and poor supply chain management [6–9]. The current pandemic adds further complexity, with health services’ shift in focus towards containing COVID-19 transmission, disruption in supply of essential medicine, burdened health facilities, poor affordability of services from private sector and fear of COVID-19 transmission while accessing the services [45].

This is one of the first studies in Nepal to explore the impact of COVID-19 pandemic on violence and SRH service access among young girls and women. However, the survey has certain limitations. The survey only included mobile phone users. Hence the survey excluded women and girls who did not have access to mobile phone. Due to the nature of the survey, it was also not possible to estimate the difference in characteristics of participants who did not complete the survey and those who did. It was not possible to apply sample weights to groups who were less likely to have access to mobile phone due to lack of data. The survey assessed prevalence of violence before and after the pandemic but did not look into the difference in frequency and intensity of violence among the victims. There is a possibility of recall bias among participants when recalling experience of violence, especially their experience before COVID-19 pandemic. Lastly, the cross-sectional nature of the study makes it difficult to establish temporal association between predictor and outcome variables.

Although the study did not find significant difference in the prevalence of violence before and after the pandemic, it reports on the correlates of violence among girls and women for both time period. Education was found to be a protective factor against violence both before and after the pandemic. This bolsters the importance of preventing education discontinuation among girls and young women during the pandemic and bringing out of school girls back into the education system. Dalit girls and women were at higher risk of violence during the pandemic. This suggests that social exclusion and subsequent disadvantages heightens the risk of violence during the pandemic and highlights a need for targeted intervention in this community. The findings also suggest a need to improve essential SRH services among girls and women with special focus on girls and women from rural municipalities and girls and women with disability.

## Supporting information

S1 Data (XLSX)

S1 File (DOCX)
