# Supplementary material for: Violence and sexual and reproductive health service disruption among girls and young women during COVID-19 pandemic in Nepal: A cross-sectional study using interactive voice response survey
Source: PLoS One. 2021 Dec 8;16(12):e0260435. doi: 10.1371/journal.pone.0260435 (PMC8654226; doi:10.1371/journal.pone.0260435)
Supplement: S1 File — (DOCX) [file pone.0260435.s002.docx]

Log of final changes:

1. Minor grammatical changes throughout the document.
2. Line 32: Corrected Odds ratio
3. Line 137: Added reference citation (no changes to the reference list required).
4. Line 165: Added a line explaining distribution of “other” SRH service disruption.
5. Line 200: Table 6, changed decimal places to 2 for the confidence interval.
6. Line 222: Added reference citation (no changes to the reference list required).
